# Mapping the ‘breaker’ element of the gametocidal locus proximal to a block of sub-telomeric heterochromatin on the long arm of chromosome 4S^sh^ of *Aegilops sharonensis*

**DOI:** 10.1007/s00122-015-2489-x

**Published:** 2015-03-08

**Authors:** Emilie Knight, Ashleigh Binnie, Tracie Draeger, Matthew Moscou, María-Dolores Rey, Justine Sucher, Surbhi Mehra, Ian King, Graham Moore

**Affiliations:** 1 Crop Genetics Department, John Innes Centre, Norwich Research Park, Colney, Norwich, NR4 7UH UK; 2 The Sainsbury Laboratory, Norwich, UK; 3 Plant Breeding Department, Institute for Sustainable Agriculture, Agencia Estatal Consejo Superior de Investigaciones Científicas (CSIC), Córdoba, Spain; 4 Institute of Plant Biology, University of Zurich, Zurich, Switzerland; 5 Division of Plant and Crop Sciences, School of Biosciences, The University of Nottingham, Sutton Bonington Campus, Loughborough, UK

## Abstract

**Key message:**

**The ‘breaker’ element (** ***GcB*** **) of the gametocidal locus derived from** ***Aegilops sharonensis*** **has been mapped to a region proximal to a block of sub-telomeric heterochromatin on chromosome 4S** ^**sh**^ **L.**

**Abstract:**

The production of alien chromosome addition lines allows the transfer of useful genetic variation into elite wheat varieties from related wild species. However, some wild relatives of wheat, particularly those within the Sitopsis section of the genus *Aegilops*, possess chromosomes that are transmitted preferentially to the offspring when addition lines are generated. Species within the Sitopsis group possess the S genome, and among these species, *Aegilops sharonensis* (2*n* = 14, S^sh^S^sh^) carries the S^sh^ genome which is closely related to the D genome of hexaploid wheat. Some S genome chromosomes carry gametocidal loci, which induce severe chromosome breakage in gametes lacking the gametocidal chromosome, and hence, result in gamete abortion. The preferential transmission of gametocidal loci could be exploited in wheat breeding, because linking gametocidal loci with important agronomic traits in elite wheat varieties would ensure retention of these traits through successive generations. In this study, we have mapped the breaker element of the gametocidal locus derived from *Ae. sharonensis* to the region immediately proximal to a block of sub-telomeric heterochromatin on the long arm of chromosome 4S^sh^.

**Electronic supplementary material:**

The online version of this article (doi:10.1007/s00122-015-2489-x) contains supplementary material, which is available to authorized users.

## Introduction

The Sitopsis section of the genus *Aegilops* comprises five diploid species that carry the S genome, which is closely related to either the B or D genome of hexaploid wheat (*Triticum aestivum*) depending on its lineage (Marcussen et al. 2014). Several of these Sitopsis species possess gametocidal (*Gc*) loci which, when introgressed into wheat (as monosomic addition lines or substitutions), cause abortion in the male and female gametes that do not have chromosomes carrying these loci. The gametocidal action on the female side results in limited seed set, while on the male side, it results in the occurrence of both normal and abortive pollen. It is easier to investigate action of these loci on the male side, and such studies reveal chromosome aberrations during first pollen mitosis in the gametes. These aberrations include chromatid fragments, dicentric chromosomes and chromatin bridges (Finch et al. 1984; King and Laurie 1993; Nasuda et al. 1998). As a consequence of these aberrations, the only gametes to survive are those carrying chromosomes with a gametocidal locus (*Gc* chromosomes), which is roughly 50 % of the pollen cells, thus ensuring the preferential transmission of these chromosomes to subsequent generations. This genetic phenomenon is of particular interest to breeders, because linking *Gc* loci with valuable traits in elite varieties would ensure maintenance of these traits in subsequent generations without the need for selection. This could be achieved by a GM approach; whereby the *Gc* locus would be present in a vector with the gene for the trait of interest (for example a disease resistance gene), which would then be inserted by transformation in the genome of the elite wheat variety. The progeny carrying this construct would always come through.

Studies of a range of different *Gc* chromosomes suggest that there are at least two phenotypes associated with the mechanism responsible for the preferential transmission of the *Gc* chromosome (Endo 1990; reviewed Tsujimoto 2005). The first phenotype is referred to as the ‘breaker’, and induces chromosome breakage. The second is an ‘inhibitor’ phenotype, which prevents chromosome breakage. Chromosome aberrations do not occur in gametes carrying both elements, as the gametocidal action is neutralised by the inhibitor. This hypothesis is supported by the observations that *T. aestivum* cv. Norin 26 possesses a suppressor of the 3C gametocidal locus derived from *Ae. caudata* (Tsujimoto and Tsunewaki 1985b), and that the development of a knock-out mutant of the breaker element *Gc2* on *Ae. sharonensis* chromosome 4S^sh^, has lost the chromosome fragmentation function, but has retained the inhibitor element (Friebe et al. 2003). The gametocidal loci within Sitopsis genomes are classified in groups according to their action and strength (Endo 1990). *Ae.* *sharonensis* carries gametocidal loci on chromosomes 2S^sh^ and 4S^sh^ (Endo 1985).

The *Ae. sharonensis* 4S^sh^ breaker element (*GcB*) has been previously mapped by C-banding to the distal end of the long arm (Endo 2007). The aim of the present study was to further map *GcB* on *Ae. sharonensis* chromosome 4S^sh^. This chromosome is homoeologous to wheat group 4. Previously, two hexaploid wheat introgression lines (Brigand 8/2 and Brigand 8/9) had been developed, involving the translocation of the long arm of chromosome 4S^sh^ (4S^sh^L) from *Ae. sharonensis* onto the long arm of chromosome 4D of *T. aestivum* (4DS·4DL-4S^sh^L translocation) (King et al. 1996). The occurrence of this translocation is consistent with recent studies showing that the *Ae. sharonensis* genome is more closely related to the D genome than the B genome (Marcussen et al. 2014). The strategy for mapping *GcB* employed a similar approach as that used for the mapping of the *Ph1* locus (Roberts et al. 1999; Griffiths et al. 2006). This involved developing irradiated populations of the introgression line, and screening these populations with DNA markers specifically designed to the 4S^sh^L chromosome segment. Traditional mapping approaches such as development of NILs (near isogenic lines) were not possible, since the *Ph1* locus present in polyploid wheat prevents recombination between wheat and homoeologous (e.g. alien) chromosomes. Irradiation of populations for mapping the *Ph1* locus generated deletions alone, but irradiation of the introgression line populations induced both deletions and several translocations of differing sizes involving the 4S^sh^L segment. These mutant lines were then phenotyped for the presence or absence of *GcB*. This phenotyping involved scoring the fertility of F_1_ hybrids generated from crossing the deletion or translocation line with wild type wheat, as well as analysing chromosome fragmentation in first pollen mitosis. The sizes of new translocations and deletions were characterized using markers for the 4S^sh^L segment, so that a minimum region covering the *GcB* element could be defined. Using this approach, the *GcB* element was delimited to a small region immediately proximal to a sub-telomeric heterochromatin block at the distal end of the chromosome.

## Materials and methods

### Plant material

Two *T. aestivum* cv. ‘Brigand’ introgression lines (Brigand 8/2 and Brigand 8/9), each carrying a 4DS·4DL-4S^sh^L translocation from *Ae. sharonensis* accession 2170001 (King et al. 1996), were individually crossed with *T. aestivum* cv. ‘Huntsman’, using Huntsman as the pollen donor. Huntsman does not possess the 4DS·4DL-4S^sh^L translocation, so the resulting F_1_ seeds were hemizygous for the *Ae. sharonensis* introgression. Two mutant populations were created by gamma irradiation. A total of 1011 hybrid F_1_ (M_0_) seeds were irradiated with 300 Grays (558 from the Brigand 8/2 × Huntsman cross and 453 from the Brigand 8/9 × Huntsman cross) and 586 hybrid F_1_ (M_0_) seeds were irradiated with 350 Grays (395 from the Brigand 8/2 × Huntsman cross and 191 from the Brigand 8/9 × Huntsman cross). The M_1_ seeds irradiated with 300 Grays and 350 Grays germinated at a rate of 87.8 % (personal communication, Surbhi Mehra) and 49 %, respectively. Spikes were semi-sterile, which confirmed the effect of the gametocidal locus in these hemizygous plants. M_1_ plants were self-pollinated to give homozygous M_2_ seeds. M_2_ plants were screened for deletions and/or chromosomal translocations. Lines with deletions or chromosomal translocations in the *Ae. sharonensis* introgression from the Y300 population were backcrossed onto *T. aestivum* cv. Huntsman to check for the presence or absence of the gametocidal effect. Deletion and translocation lines 5F8, 5D12, 7C12, 12A5, and 12H3 all originated from an initial cross between introgression line Brigand 8/2 and *T. aestivum* cv. Huntsman.

### Marker development

Thirty-three markers were designed that showed polymorphism between *T. aestivum* cv. Huntsman and the introgressed *Ae. sharonensis* segment from accession 2170001 in Brigand 8/2 and Brigand 8/9. Nine of these markers were initially designed as SSCP–SNP markers (Bertin et al. 2005) from *Ae. sharonensis* accession 1644 sequences obtained from a transcriptome assembly by Bouyioukos et al. (2013). These were aligned to their corresponding wheat homologs based on a best BLAST hit to predict intron position and size (Brenchley et al. 2012). When wheat sequence was not available, the rice (*Oryza sativa*) homolog was used. These SSCP–SNP markers were later converted into high-throughput KASP™ markers (He et al. 2014), by designing triplets of FAM, VIC and common primers around SNPs between *T. aestivum.* cv. Huntsman and the *Ae. sharonensis* accession 2170001 allele. Each marker was given the prefix ‘*KASP*’ followed by the *Ae. sharonensis* cDNA read number from the transcriptome analysis.

Marker *UTV39* was designed from nucleotide sequence of clone UTV39 (Giorgi et al. 2003), forward primer 5′GATCCGTTTGGTCATCAGGT and reverse primer 5′GATCTTCCAGTAGTTCCTAA. This marker was later converted into a KASP™ marker as for the SSCP–SNP markers and was designated as ‘*KASP UTV3*’ (Online Resource 1).

The remaining twenty-four markers were obtained from two analyses using the high density 90,000 SNP genotyping array (Wang et al. 2014). The first analysis identified KASP™ markers with polymorphisms between the genomic DNA of Brigand with no introgression and Brigand 8/2 amongst the 90,000 SNPs of the array. These markers identified polymorphisms between the *Ae.* *sharonensis* introgression and wheat group 4 chromosomes. The second analysis was carried out using genomic DNA from Huntsman and the Y300 translocation line 12A5 to identify further markers in the distal region of the *Ae. sharonensis* introgressed segment. These twenty-four markers were each designated with a unique eight digit number.

Twenty-two of the thirty-three KASP™ markers were further checked for specificity to the D genome, and redesigned when necessary to amplify the wheat D allele only (VIC primer) and the *Ae. sharonensis* allele (FAM primer). *Ae.* *sharonensis* sequence was obtained by BLASTing the SNP sequence to the *Ae. sharonensis* whole-genome database (Marcussen et al. 2014). The *Ae.* *sharonensis* sequence was then aligned to the corresponding A, B and D best BLAST hits obtained by BLASTing the SNP sequence to 4A, 4B, 4D and 5A on the URGI BLAST webpage (https://urgi.versailles.inra.fr/blast/blast.php) for each marker. These twenty-two D genome specific KASP™ markers were used to genotype M_2_ plants (Online Resource 1).

*KASP* *UTV39* could not be designed as a D specific marker as it is a repeat sequence specific to the S^sh^ genome and has no significant BLAST hit to a D sequence in wheat. The best wheat BLAST hit is a 5BS contig.

### Genotyping of Y300 M_2_ population

DNA extractions were carried out on leaf material of M_2_ plants at the 2–3 leaf stage, according to the Somers and Chao protocol http://maswheat.ucdavis.edu/PDF/DNA0003.pdf original reference in Pallotta et al. (2003).

PCR reactions for SSCP–SNP markers were carried out using a touchdown program: 95 °C for 15 min, then ten cycles of 95 °C for 30 s, 63 °C for 30 s (−0.5 °C per cycle) and 72 °C for 90 s, followed by 30 cycles of 95 °C for 30 s, 58 °C for 30 s and 72 °C for 90 s. NB: for marker *UTV39*, the annealing temperature was 50 °C instead of 58 °C. Markers were run on SSCP gels as described in Bertin et al. (2005).

All KASP™ amplifications were carried out using 2 µl of KASP™ master mix (LGC group, United Kingdom), 0.056 µl of primer mix (12 µl FAM primer at 100 µM + 12 µl of VIC primer at 100 µM + 30 µl of common primer at 100 µM + 46 µl of dH_2_O) and 2 µl of DNA at 5 ng/µl, following the same program with an optimal cycle number varying from 30 to 40 for each marker (Online Resource 1): 94 °C for 15 min, 10 cycles of 94 °C for 20 s, 65 °C for 1 min and 94 °C for 20 s, then either 30, 35 or 40 cycles of 94 °C for 20 s and 57 °C for 1 min. Fluorescence was measured by a Tecan Safire plate reader. Results were analysed using the KlusterCaller software (LGC group, UK).

### Fluorescence in situ hybridisation (FISH)

Root tip squashes from germinating seedlings of *T. aestivum* cv. Brigand 8/2 and M_3_ seedlings of lines 12A5, 12H3 and 7C12 were prepared as described in Gill et al. (1991) with minor modifications.

Fluorescence in situ hybridisation (FISH) was carried out as described in Prieto et al. (2007). The probe used was a dUTP-digoxigenin labelled product generated by amplification of high quality DNA of *Ae. sharonensis* accession 1644 with *UTV39* primers. Labelling with dUTP-digoxigenin was carried out directly, with no nick translation being necessary due to the short length of the PCR product. Observations were made using a Nikon Eclipse E600 CCD microscope and images captured with a Hamamatsu ORCA-ER C4742-95 digital camera. Images were merged using ImageJ software.

### Phenotyping

The phenotype of each M_2_ hybrid plant was determined by measuring spike fertility and by looking at chromosome fragmentation during first pollen mitosis. Spike fertility was measured as the percentage of florets that contained well-developed seeds. Only the two outermost (oldest) florets within each spikelet were counted. Undersized florets at the top and bottom of the spike were not included in the calculations. Hybrid plants were grown in three batches, with a 2 week interval between each sowing. One to four plants were grown for each line per batch and three to eight ears per plant were phenotyped. Parental plants of Huntsman and Brigand 8/2 were grown as controls alongside each batch so that environmental effects on spike fertility could be taken into account. The average percentage seed set was calculated from 102, 82 and 78 phenotyped ears of combined Huntsman and Brigand 8/2 plants for batches 1, 2 and 3, respectively. The average percentage of viable seeds was calculated from all phenotyped ears for each hybrid line [7C12, 12A5, 12H3 and whole segment translocation line (WST), backcrossed onto Huntsman], per batch. Each average was adjusted according to the average percentage of viable seeds for self-pollinated parental lines used as controls calculated per batch, to take into account any environmental factors. This was carried out by multiplying the mutant hybrid line average by 100 and then dividing it by the average for the controls. An average of approximately 50 % viable seeds indicated that *GcB* was present as it lead to abortion of around half of the gametes.

Observations of chromosome fragmentation at anaphase of first pollen mitosis were carried out on aceto-carmine stained anther squashes, using a Leica DM2000 microscope and a Leica DFC450 image capture system.

## Results

### Marker development

Thirty-three markers were designed to be polymorphic between hexaploid wheat and the 4S^sh^L *Ae. sharonensis* segment to map the gametocidal breaker element (*GcB*) within this introgression. Twenty-two of these markers were further designed to be specific to the hexaploid wheat D genome and polymorphic with the orthologous 4S^sh^ sequence.

**Fig. 1:**
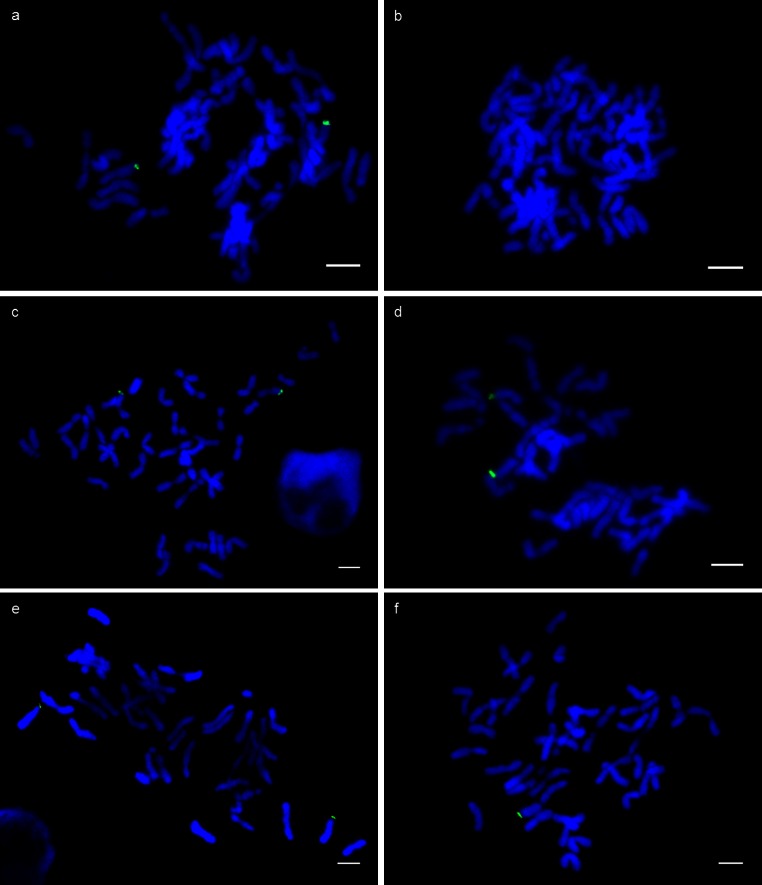
Fluorescence in situ hybridisation using probe UTV39 (*green*) on DAPI stained chromosomes of mitotic cells from root tip squashes of parental lines Brigand 8/2 (**a**) and Huntsman (**b**), translocation lines 12A5 (**c**), 12H3 (**d**) and deletion line 7C12 type 1 (**e**) and type 2 (**f**). *Scale bar* represents 10 µm

The terminal region of the 4S^sh^L chromosome segment carries a large block of heterochromatin. Probe pAesKB52 has been shown to hybridise to major sub-telomeric heterochromatin sites in *Ae. sharonensis* (Anamthawat-Jónsson and Heslop-Harrison 1993) and its sequence shares 90.1 % identity to clone UTV39 (Giorgi et al. 2003). This indicated that the UTV39 repeat would constitute a good probe for the detection of the block of sub-telomeric heterochromatin present on 4S^sh^L. This was confirmed by a FISH experiment using the UTV39 repeat as a probe on mitotic cells from root tip squashes of 4DS·4DL-4S^sh^L Brigand 8/2 translocation line (Fig. 1a). As expected, the probe hybridised to the distal end of two chromosomes in each cell (chromosomes 4D carrying the 4S^sh^ introgression). The UTV39 repeat could not be converted to a D genome specific marker for the genotype screens as it is specific to the S genome, but could be used as a presence/absence marker for the S copies of the UTV39 repeat. Orthologous sequences of this repeat are distributed throughout the hexaploid wheat genome, but these are in relatively small numbers compared to the estimated 40,000 copies present on four of the seven chromosomes of *Ae. sharonensis* (Giorgi et al. 2003). Fluorescence in situ hybridisation (FISH) experiments using the UTV39 repeat as a probe on hexaploid wheat root tips do not detect a signal (as shown in Fig. 1b: FISH using UTV39 probe on root tip squashes of Huntsman), but when UTV39 is used as an SSCP–SNP marker or in KASP™ technology, an amplified product is generated both for hexaploid wheat (e.g. cv. Huntsman) and *Ae. sharonensis* DNA. These products are polymorphic. However, only the S^sh^ copies of UTV39 are amplified, due to the extremely high copy number of UTV39 repeats, in translocation lines such as Brigand 8/2 and Brigand 8/9 where both the *Ae. sharonensis* (S^sh^) copies and the wheat orthologous sequences are present.

**Fig. 2:**
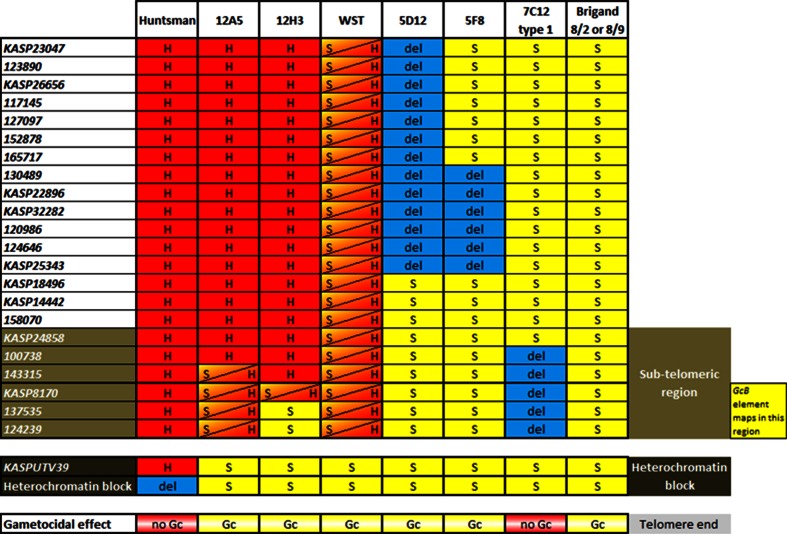
Genotypes of parental lines *T. aestivum* cv. Huntsman and 4DS·4DL-4S^sh^L translocation lines Brigand 8/2 and 8/9; translocation lines 12A5, 12H3 and the forty-eight Y300 + two Y350 whole segment translocation (WST) lines; and deletion lines 5D12, 5F8 and 7C12 type 1 using the twenty-two KASP™ markers and *KASP UTV39*. ‘*H*’ represents the Huntsman allele, ‘*S*’ the *Ae. sharonensis* allele, ‘*S/H*’ a duplication of both S and H sequence for that marker, and ‘*del*’ the absence of an amplification product, indicative of a deletion for that marker. The phenotype for each line is represented as ‘gametocidal effect’, where the breaker element is either present (*Gc*) or absent (no *Gc*). The heterochromatin block is present in *every line* except euploid wheat Huntsman. The sub-telomeric region proximal to the heterochromatin block is* highlighted*, as well as the region in which *GcB* maps

Synteny between Triticeae chromosome group 4, *Brachypodium* *distachyon* chromosome 1, and rice chromosome 3 was used as a tool to provide an approximate gene content of this 4S^sh^L terminal region. Approximately half the markers for the introgressed segment were identified by this approach. The remaining markers were derived from other sources (as described in “Materials and methods”). However, it was apparent that the order of genes is extensively rearranged within the terminal regions. This is consistent with the reported decrease in synteny levels in the distal regions of chromosomes (Akhunov et al. 2003). Consequently, the newly developed markers could not be ordered according to synteny. However, seven of the twenty-two KASP™ markers were converted into *Ae. sharonensis* specific KASP™ markers, designed around SNPs between accessions 1644 and 2189 (Bouyioukos et al. 2013). These markers were used to screen an F_6_ recombinant population resulting from a cross between *Ae. sharonensis* accessions 1644 and 2189 (resistant and susceptible to race Ug99 of stem rust pathogen, respectively). This allowed the mapping of these seven markers relative to one another (highlighted in yellow in Online Resource 1). This mapping revealed *KASP23047* as the most proximal of these seven markers. The order of all twenty-two KASP™ markers was established later in the study from the analysis of the presence or absence of these markers observed on the deleted and translocated chromosomes following irradiation (Fig. 2), with the UTV39 repeat being the most distal marker.

### Screening of irradiated populations

**Fig. 3:**
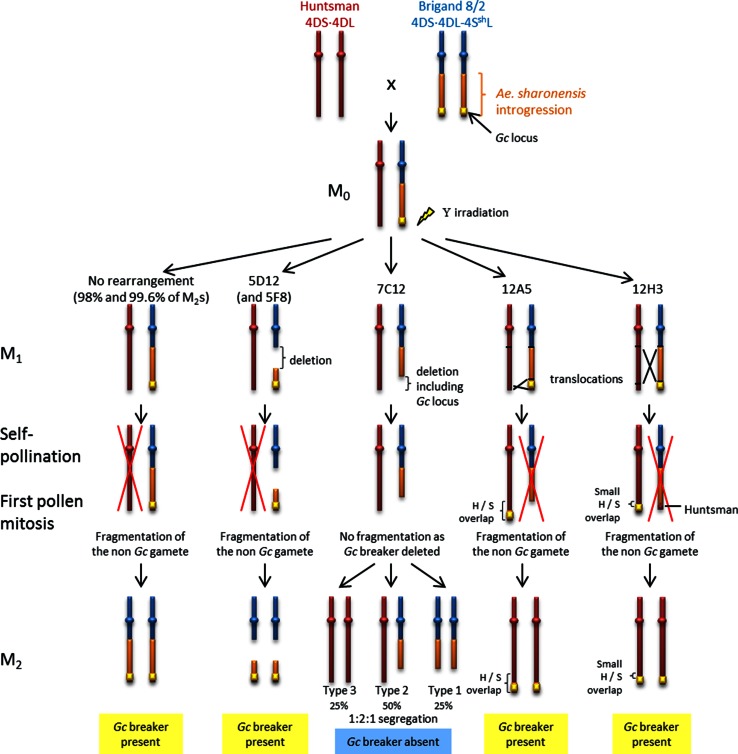
Diagram representing chromosomal rearrangements caused by gamma irradiation in lines 5D12 (and 5F8—similar segregation pattern but different size deletion), 7C12, 12A5 and 12H3 and their segregation from M_1_ to M_2_ plants. *Lines with no rearrangement* in the *Ae. sharonensis* introgression are also represented and constituted 98 and 99.6 % of the Y300 and Y350 populations, respectively

A total of 3,764 M_2_ plants from the Y300 irradiated population and 3300 M_2_ plants from the Y350 irradiated population were genotyped using the twenty-two KASP™ markers and *KASP UTV39* (Online Resource 1). Ninety-eight percent of the Y300 M_2_ plants and 99.6 % of the Y350 M_2_ plants carried just the Brigand 4DS·4DL-4S^sh^L translocated chromosome, possessing a homozygous S (*Ae. sharonensis* SNP allele) genotype. Thus, the Brigand 4DS·4DL-4S^sh^L translocated chromosome is preferentially transmitted, while the 4D (Huntsman) chromosome lacks the introgression and therefore, the *Gc* locus is fragmented. The H (Huntsman SNP) allele is not present in most M_2_ plants (Fig. 3).

#### Deletions lines resulting from the Y350 irradiation

The genotype screening of M_2_ plants derived from the Y350 irradiated M_1_ population, identified three lines carrying deletions involving the 4S^sh^L segment. However, genotyping revealed that the whole *Ae. sharonensis* segment had been deleted in one of these lines, so *GcB* could not be further mapped using this line. The remaining two deletion lines (5F8 and 5D12) derived from the Y350 population possessed a deletion encompassing six markers (*130489*–*KASP25343*), and thirteen markers (*KASP23047*–*KASP25343*), respectively within the introgressed segment (Fig. 2). Both these M_2_ deletion lines are a homozygous S genotype for the remaining *Ae. sharonensis* alleles. The absence of Huntsman alleles in these lines indicated that the Huntsman chromosome had been fragmented at the M_1_ stage (Fig. 3), and so the *Gc* locus is still present in both the non-deleted segments of the *Ae. sharonensis* introgression, i.e. within the region from the telomere to marker *KASP25343*.

#### Translocation lines resulting from the Y350 irradiation

**Fig. 4:**
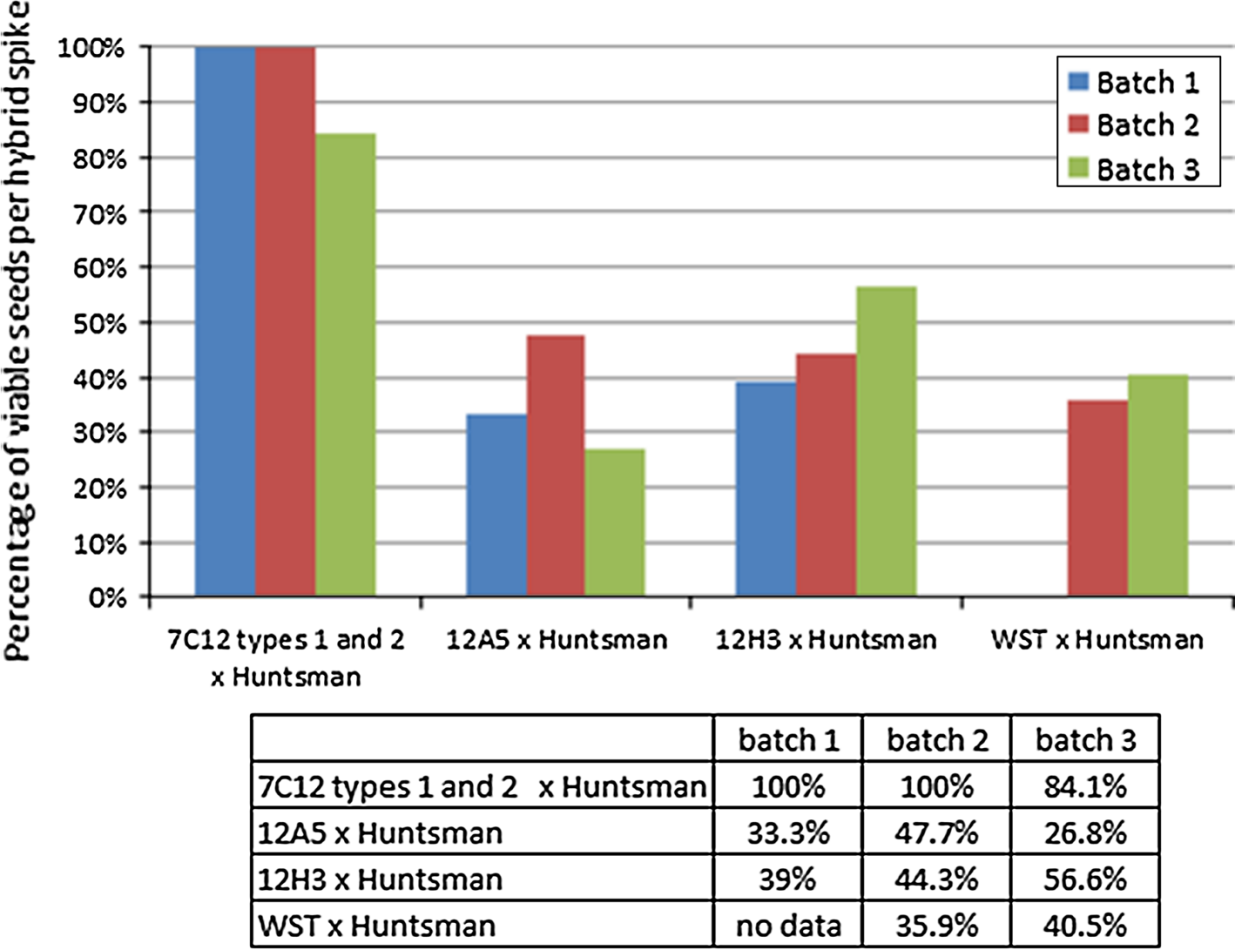
Percentage of viable seeds in spikes of hybrid plants resulting from the backcross of deletion line 7C12 (types 1 and 2 combined) and translocation lines 12A5, 12H3 and the WST lines onto wheat cultivar Huntsman. An average percentage was calculated for each hybrid type. Three batches of plants were sown, except for the WST lines for which only two batches were sown. Percentages were adjusted according to the average spike fertility for self-pollinated* parental lines* for each batch to take environmental conditions into account

The genotype screening of M_2_ plants from the Y350 irradiated M_1_ population identified two lines with chromosomal rearrangements typical of translocations. However, genotyping revealed that translocations were far more common in the Y300 population, with fifty-two lines being identified. Forty-eight of the fifty-two lines derived from the Y300 population and the two translocation lines from the Y350 population carried the whole *Ae. sharonensis* introgression translocated onto the Huntsman 4DL chromosome, such that each marker along this translocated chromosome amplified an H and an S sequence. These lines are designated as whole segment translocations (WST) (Fig. 2). The translocation did not result in the deletion of *GcB*, as the percentage of viable seeds in hybrids resulting from the backcross of these M_2_ lines onto wheat cv. Huntsman ranged from 35.9 to 40.5 % (Fig. 4), indicating that the *GcB* element was still present (percentage of seed set in self-pollinated spikes of parental Huntsman and introgression line Brigand 8/2 was also determined to take account of any environmental impact on seed viability). The reciprocal product of this event, a truncated Brigand 8/2 chromosome, would have been fragmented by the *GcB* element present in the other gametes and would therefore not have come through in the M_2_ generation.

**Fig. 5:**
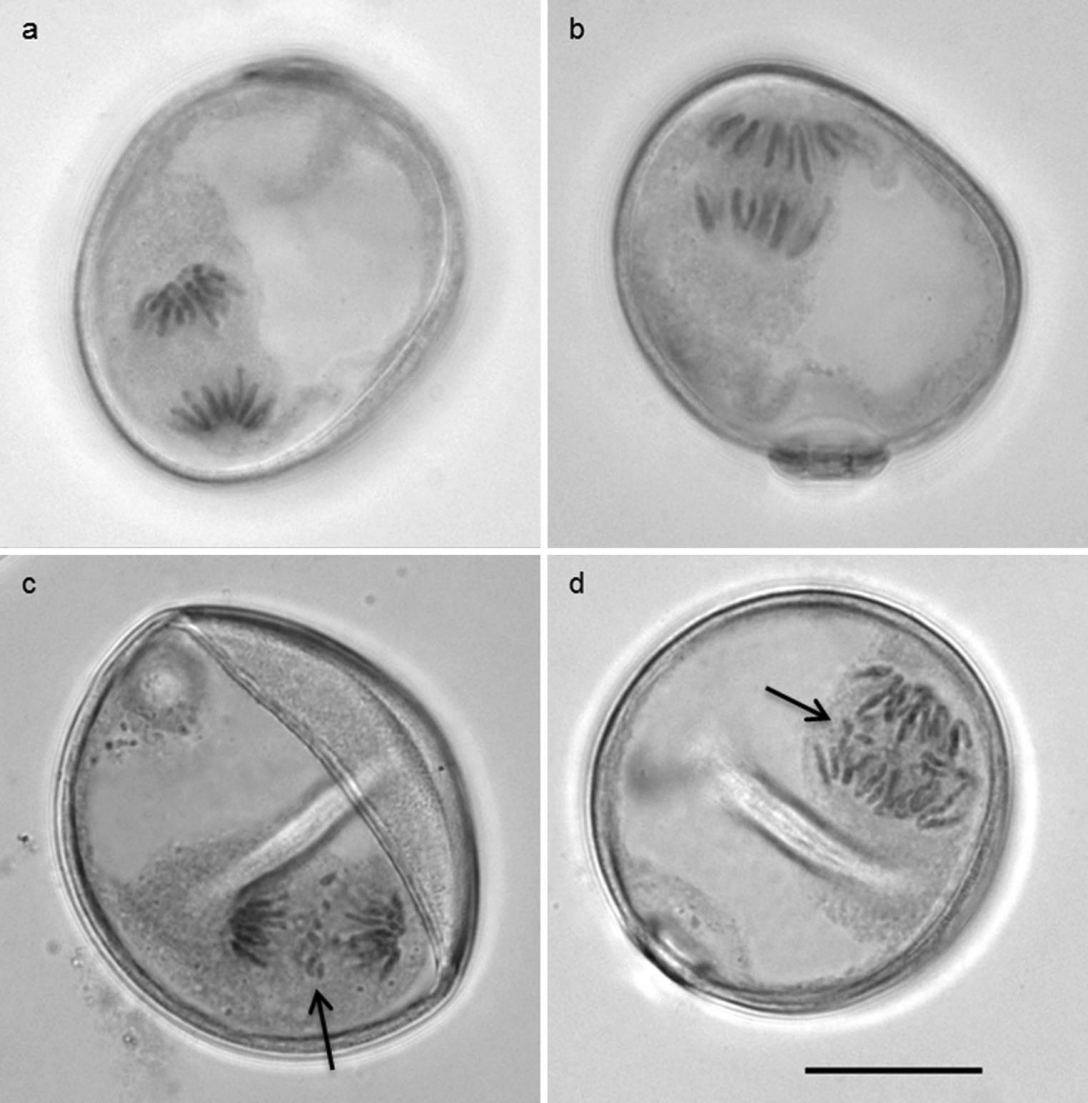
Mitotic anaphase I in pollen cells of self-pollinated wheat cv. Huntsman (**a**, **b**) and hybrid ears of a 12H3 × Huntsman backcross (**c**, **d**). Chromosome fragments caused by the *GcB* element can be seen in the hybrid cells (**c**, **d**). *Scale bar* represents 20 µm

Of the remaining four translocation lines (12A5, 12H3, 12H4 and 12H5) derived from the Y300 population, three (12H3, 12H4 and 12H5) originated from the same M_1_ ear and shared the same translocated genotype. Thus, only one of these lines, 12H3, is represented in Figs. 2, 3 and 4. Line 12A5 (Fig. 2) has a translocation of the distal region of the *Ae. sharonensis* segment (comprising markers *143315*, *KASP8170*, *137535* and *124239*) onto the end of the Huntsman chromosome resulting in the duplication of this terminal region. Thus, markers were genotyped as both H and S as in a hemizygous situation (Figs. 2, 3). Line 12H3 has undergone a similar translocation event, with a duplication of H and S segments for marker *KASP8170* only. However, in 12H3 the two distal markers, *137535* and *124239*, were genotyped as S only (Fig. 2), which may suggest that the equivalent region of the Huntsman chromosome was translocated onto the other chromosome carrying most of the *Ae. sharonensis* introgression. This translocated chromosome lacked the *Gc* locus and would have therefore been fragmented during pollen mitosis of the M_1_ self-pollination stage (Fig. 3). The two translocation lines 12A5 and 12H3 were phenotyped by backcrossing the M_2_ plants onto Huntsman and assessing the percentage of viable seeds in the resulting hybrid ears as described for the deletion lines. Hybrid ears exhibited 26.8–47.7 % viable seeds for the 12A5 backcross and 39–56.6 % viable seeds for the 12H3 backcross (Fig. 4), indicating the presence of the gametocidal effect in both lines. Further confirmation that the semi-sterile spike phenotype resulted from the presence of the *Gc* locus in line 12H3 (which carried the smallest *Ae. sharonensis* segment) was obtained by observing fragmentation in half the pollen cells of hybrid plants resulting from the 12H3 backcross (Fig. 5). Numerous chromosome fragments were observed between separating chromatids at anaphase of first pollen mitosis in the 12H3 backcrossed onto Huntsman hybrids, typical of previous observations of gametocidal induced fragmentation (Finch et al. 1984; King and Laurie 1993; Nasuda et al. 1998). This provided evidence that the *GcB* element was still present in this line.

These results, and especially the analysis of the smallest introgression segment (line 12H3), further defined the *GcB* element as being between marker *143315* and the telomere including the heterochromatin block on the *Ae. sharonensis* segment (Fig. 2). Moreover, the 4S^sh^L segment in 12H3 may also contain the inhibitor element. This translocated segment causes fragmentation of chromosomes lacking the 4S^sh^L sub-telomeric segment but not of itself, suggesting that it also carries the inhibitor element. However, this would need to be confirmed by independent mapping of this element.

#### Deletions lines resulting from the Y300 irradiation

The genotype screening of M_2_ plants from the Y300 irradiated M_1_ population also identified nine lines carrying deletions involving the 4S^sh^L segment. As in the case of those identified in the Y350 irradiated M_1_ population, the whole *Ae. sharonensis* segment had been deleted in eight of these lines. However, the remaining line, 7C12, had a deletion of only part of the 4S^sh^L segment. Genotyping analysis of this line revealed that the M_2_ progeny segregated with a 1:2:1 inheritance ratio (Fig. 3). Genotyping identified 25 % of the progeny as having the Huntsman allele only (type 3) and 50 % as having both S and H alleles for most of the introgressed segment, with the region from *100738* to *124239* being Huntsman only (type 2). The lack of fragmentation of the Huntsman chromosome suggested that the *GcB* element must have been deleted in the M_1_ line as a result of the irradiation. This gave rise to the three progeny types illustrated in Fig. 3. The remaining 25 % of the M2 progeny for 7C12 (type 1) were homozygous for a partially deleted Brigand 4DS·4DL-4S^sh^L translocated chromosome (deletion covering markers *100738*–*124239*).

The presence of the *GcB* element was confirmed by backcrossing plants homozygous for the partially deleted Brigand 4DS·4DL-4S^sh^L translocated chromosome (7C12 type 1) and hemizygous for the partially deleted Brigand 4DS·4DL-4S^sh^L translocated chromosome and the Huntsman 4D chromosome (7C12 type 2) onto the Huntsman parent. Hybrid plants resulting from the backcrosses were left to mature and the percentage of viable seeds was calculated for each ear. Spikes from hybrid plants resulting from crosses between 7C12 M_2_ types 1 and 2 onto wheat cultivar Huntsman had a combined average of 84.1–100 % viable seeds (Fig. 4), confirming the absence of fragmentation at the M_1_ stage.

Genotyping revealed that the 4S^sh^L segment in 7C12 carried a deletion involving the very distal region from markers *100738* to *124239*. However, surprisingly, genotyping also revealed that this line carried S copies of the UTV39 repeat sequence, suggesting that the introgressed segment may still carry the *Ae. sharonensis* heterochromatin block and telomere region. Confirmation of this required FISH analysis using the *UTV39* marker as a probe, on root tip squashes derived from this line to confirm the presence of the heterochromatin block. *KASP UTV39* and SSCP–SNP *UTV39* genotyping revealed all six lines (5F8, 5D12, 7C12 types 1 and 2, 12A5 and 12H3) to carry S copies of the UTV39 repeat sequence (Fig. 2). FISH on mitotic cells from root tip squashes of lines 12A5, 12H3, 7C12 type 1 and 7C12 type 2 (Fig. 1c–f) mirrored the genotyping analysis, revealing the presence a hybridisation signal for probe UTV39 in all four lines. The hybridisation signal observed in cells of lines 12A5, 12H3 and 7C12 type 1 was identical to that observed in the Brigand 8/2 parental line (Fig. 1a) and in Giorgi et al. (2003); i.e. a hybridisation signal at the telomeric end of two of the chromosomes (the 4D pair). Line 7C12 type 2 is the hemizygous genotype and therefore only has one chromosome carrying the S copies of *UTV39*, which is observed as a hybridisation signal at the end of only one chromosome (Fig. 1f). The presence of the heterochromatin block is expected for lines 5F8, 5D12, 12A5 and 12H3, as the distal markers in the 4S^sh^L region are present. However, it is surprising that these same markers are deleted in 7C12, yet the heterochromatin block is retained. Thus, the analysis reveals that the *GcB* element must map in the region immediately proximal to the heterochromatin block.

## Discussion

The *GcB* element of the *Gc* locus has thus been mapped to the region distal to marker *143315* on chromosome 4S^sh^L (Fig. 2) and proximal to the heterochromatin block. Marker *KASP24858* maps proximal to marker *143315* (Fig. 2), and is orthologous to rice gene LOC_Os03g02600 (Online Resource 1). Yet, this gene is present on wheat BAC_2050O8 which has been localised to the telomeric bin 4BL-10 (0.95–1.0) (Salina et al. 2009). This BAC also carries copies of the Spelt 52 repeat in two arrays in a “head-to-tail” orientation (Salina et al. 2009). Spelt52 is analogous to pAesKB52 (Salina et al. 2006) which in turn is analogous to UTV39 (Giorgi et al. 2003). This suggests that marker *KASP24858* is sub-telomeric, along with the other five markers distal to it (*100738*–*124239*) down to the heterochromatin block in a similar structure to that of BAC_2050O8 (Salina et al. 2009). Consequently, the *GcB* element is defined to the region immediately proximal to the sub-telomeric heterochromatin block (represented in Fig. 2) and might constitute a very small region.

The localisation of the *GcB* element in the region proximal to the heterochromatin block will enable further mapping of the area and identification of the *GcB* element. One strategy would be to screen the wheat cultivar Chinese spring BAC library with markers *143315*, *KASP8170*, *137535* and *124239* to physically contig this region, enabling its size to be estimated. The corresponding *Ae. sharonensis* sequences could then be identified from the *Ae. sharonensis* whole-genome database (Marcussen et al. 2014) and aligned to form an *Ae.* *sharonensis* contig from which the *GcB* element could be fine mapped. This strategy could be combined with undertaking RNA-Seq on pollen cells derived from the lines carrying different translocated and deleted chromosomes. Candidates for the locus would be anything expressed in the pollen cells from 5F8, 5D12, 12A5 and 12H3, but absent in 7C12.

Several hypotheses have been proposed for the mode of action of the gametocidal locus. These include a dual-function model (breaker and inhibitor), in which the locus is similar to a restriction-modification system in bacteria (Wilson and Murray 1991; Tsujimoto 2005). However, there is one hypothesis, which is consistent with the mapping of the *GcB* element to the sub-telomeric region. Tsujimoto and Tsunewaki (1985a) suggested a transposon theory based on hybrid dysgenesis in *Drosophila melanogaster*, caused by *P* elements. The symptoms include sterility, lethality, mutations, and chromosome breakage, which are all identical to gametocidal induced symptoms. *P* elements are mobile elements. They can produce the transposase required for their mobility, which makes them autonomous. The fragmentation of chromosomes in other gametes could simply result from the activation of mobile elements by a factor which is able to travel between the gametes, while the suppressor activity is unable to travel. *P* elements can be inhibited by *P*-repressors which can be located elsewhere in the genome. This is referred to as the trans silencing effect (TSE) (Ronsseray et al. 1998). TSE could be the means by which the gametocidal inhibitor suppresses the effects of the gametocidal breaker. This hypothesis would explain the suppression by *T. aestivum* cv. Norin 26 of the *GcB* effect from *Ae. caudata* (Tsujimoto and Tsunewaki 1985b). Ronsseray et al. (1998) reported a pair of autonomous *P* elements inserted at the telomere of an X chromosome in *D. melanogaster*, in telomeric associated sequences (TAS). This model is a good hypothesis for the gametocidal system: the breaker may be a transposon similar to these telomeric *P* elements and the inhibitor would be located close to the breaker in the sub-telomeric region. The mapping of the *GcB* to this chromosome region is consistent with this hypothesis for the mode of action of the gametocidal locus.

### **Author contribution statement**

EK wrote the article, designed markers and carried out the cytological analyses. EK and TD designed the experiments, developed and processed plant populations and collected and analysed data. TD and GM revised the article. MM performed the analysis of the *Ae. sharonensis* transcriptome, developed a genetic map providing a core order of the markers and advised on marker design. AB, MDR and JS assisted with marker development, screen of M_2_ populations and phenotyping of deletion and translocation lines. SM produced the Y300 population and grew and harvested the M_1_. GM and IK designed the project and obtained funding. GM supervised experimental designs and data analyses.

## Electronic supplementary material

Below is the link to the electronic supplementary material.

Supplementary material 1 (XLSX 79 kb)

## References

[CR1] Akhunov ED, Akhunova AR, Linkiewicz AM, Dubcovsky J, Hummel D, Lazo GR, Chao SM, Anderson OD, David J, Qi LL, Echalier B, Gill BS, Miftahudin Gustafson JP, La Rota M, Sorrells ME, Zhang DS, Nguyen HT, Kalavacharla V, Hossain K, Kianian SF, Peng JH, Lapitan NLV, Wennerlind EJ, Nduati V, Anderson JA, Sidhu D, Gill KS, McGuire PE, Qualset CO, Dvorak J (2003). Synteny perturbations between wheat homoeologous chromosomes caused by focus duplications and deletions correlate with recombination rates. Proc Natl Acad Sci USA.

[CR2] Anamthawat-Jónsson K, Heslop-Harrison JS (1993). Isolation and characterization of genome-specific DNA-sequences in *Triticeae* species. Mol Gen Genet.

[CR3] Bertin I, Zhu JH, Gale MD (2005). SSCP-SNP in pearl millet—a new marker system for comparative genetics. Theor Appl Genet.

[CR4] Bouyioukos C, Moscou MJ, Champouret N, Hernandez-Pinzon I, Ward ER, Wulff BBH (2013). Characterisation and analysis of the *Aegilops sharonensis* transcriptome, a wild relative of wheat in the Sitopsis Section. PLoS One.

[CR5] Brenchley R, Spannagl M, Pfeifer M, Barker GLA, D’Amore R, Allen AM, McKenzie N, Kramer M, Kerhornou A, Bolser D, Kay S, Waite D, Trick M, Bancroft I, Gu Y, Huo N, Luo MC, Sehgal S, Gill B, Kianian S, Anderson O, Kersey P, Dvorak J, McCombie WR, Hall A, Mayer KFX, Edwards KJ, Bevan MW, Hall N (2012). Analysis of the breadwheat genome using whole-genome shotgun sequencing. Nature.

[CR6] Endo TR (1985). Two types of gametocidal chromosome of *Aegilops sharonensis* and *Aegilops longissima*. Jpn J Genet.

[CR7] Endo TR (1990). Gametocidal chromosomes and their induction of chromosome mutations in wheat. Jpn J Genet.

[CR8] Endo TR (2007). The gametocidal chromosome as a tool for chromosome manipulation in wheat. Chromosom Res.

[CR9] Finch RA, Miller TEA, Bennett MD (1984). “Cuckoo” *Aegilops* addition chromosome in wheat ensures its transmission by causing chromosome breaks in meiospores lacking it. Chromosoma.

[CR10] Friebe B, Zhang P, Nasuda S, Gill BS (2003). Characterization of a knock-out mutation at the *Gc2* locus in wheat. Chromosoma.

[CR11] Gill BS, Friebe B, Endo TR (1991). Standard karyotype and nomenclature system for description of chromosome bands and structural-aberrations in wheat (*Triticum aestivum*). Genome.

[CR12] Giorgi D, D’Ovidio R, Tanzarella OA, Ceoloni C, Porceddu E (2003). Isolation and characterization of S genome specific sequences from *Aegilops* sect. *sitopsis* species. Genome.

[CR13] Griffiths S, Sharp R, Foote TN, Bertin I, Wanous M, Reader S, Colas I, Moore G (2006). Molecular characterization of *Ph1* as a major chromosome pairing locus in polyploid wheat. Nature.

[CR14] He C, Holme J, Anthony J (2014). SNP genotyping: the KASP assay. Methods Mol Biol.

[CR15] King IP, Laurie DA (1993). Chromosome damage in early embryo and endosperm development in crosses involving the preferentially transmitted 4S^1^ chromosome of *Aegilops sharonensis*. Heredity.

[CR16] King IP, Cant KA, Law CN, Worland AJ, Orford SE, Reader SM, Miller TE (1996). An assessment of the potential of 4DS·4DL-4s(l)L translocation lines as a means of eliminating tall off types in semi-dwarf wheat varieties. Euphytica.

[CR17] Marcussen T, Sandve SR, Heier L, Spannagl M, Pfeifer M, Jakobsen KS, Wulff BBH, Steuernagel B, Mayer KFX, Olsen OA, Sequencing IWG (2014). Ancient hybridizations among the ancestral genomes of bread wheat. Science.

[CR18] Nasuda S, Friebe B, Gill BS (1998). Gametocidal genes induce chromosome breakage in the interphase prior to the first mitotic cell division of the male gametophyte in wheat. Genetics.

[CR19] Pallotta MA, Asayama S, Reinheimer JM, Davies PA, Barr AR, Jefferies SP, Chalmers KJ, Lewis J, Collins HM, Roumeliotis S, Logue SJ, Coventry SJ, Lance RCM, Karakousis A, Lim P, Verbyla AP, Eckermann PJ (2003). Mapping and QTL analysis of the barley population Amagi Nijo × WI2585. Aust J Agric Res.

[CR20] Prieto P, Moore G, Shaw P (2007). Fluorescence in situ hybridisation on vibratome sections of plant tissues. Nat Protoc.

[CR21] Roberts MA, Reader SM, Dalgliesh C, Miller TE, Foote TN, Fish LJ, Snape JW, Moore G (1999). Induction and characterisation of *Ph1* wheat mutants. Genetics.

[CR22] Ronsseray S, Marin L, Lehmann M, Anxolabehere D (1998). Repression of hybrid dysgenesis in *Drosophila melanogaster* by combinations of telomeric *P*-element reporters and naturally occurring *P* elements. Genetics.

[CR23] Salina EA, Lim KY, Badaeva ED, Shcherban AB, Adonina IG, Amosova AV, Samatadze TE, Vatolina TY, Zoshchuk SA, Leitch AR (2006). Phylogenetic reconstruction of *Aegilops* section *Sitopsis* and the evolution of tandem repeats in the diploids and derived wheat polyploids. Genome.

[CR24] Salina EA, Sergeeva EM, Adonina IG, Shcherban AB, Afonnikov DA, Belcram H, Huneau C, Chalhoub B (2009). Isolation and sequence analysis of the wheat B genome subtelomeric DNA. BMC Genom.

[CR25] Tsujimoto H (2005) Gametocidal genes in wheat as the inducer of chromosome breakage. Frontiers of Wheat Bioscience Memorial Issue, Wheat Information Service:33–48

[CR26] Tsujimoto H, Tsunewaki K (1985). Hybrid dysgenesis in common wheat caused by gametocidal genes. Jpn J Genet.

[CR27] Tsujimoto H, Tsunewaki K (1985). Gametocidal genes in wheat and its relatives. II. Suppressor of the 3C gametocidal gene of *Aegilops triuncialis*. Can J Genet Cytol.

[CR28] Wang SC, Wong DB, Forrest K, Allen A, Chao SM, Huang BE, Maccaferri M, Salvi S, Milner SG, Cattivelli L, Mastrangelo AM, Whan A, Stephen S, Barker G, Wieseke R, Plieske J, Lillemo M, Mather D, Appels R, Dolferus R, Brown-Guedira G, Korol A, Akhunova AR, Feuillet C, Salse J, Morgante M, Pozniak C, Luo MC, Dvorak J, Morell M, Dubcovsky J, Ganal M, Tuberosa R, Lawley C, Mikoulitch I, Cavanagh C, Edwards KJ, Hayden M, Akhunov E, Int Wheat Genome S (2014). Characterization of polyploid wheat genomic diversity using a high-density 90,000 single nucleotide polymorphism array. Plant Biotechnol J.

[CR29] Wilson GG, Murray NE (1991). Restriction and modification systems. Annu Rev Genet.

